# A Novel Bradycardia-Associated Variant in *HCN4* as a Candidate Modifier in Type 3 Long QT Syndrome: Case Report and Deep In Silico Analysis

**DOI:** 10.3390/biomedicines13041008

**Published:** 2025-04-21

**Authors:** Anna A. Bukaeva, Anastasia V. Blokhina, Maria S. Kharlap, Marija Zaicenoka, Evgenia D. Zotova, Anna V. Petukhova, Elizaveta V. Garbuzova, Anastasia A. Zharikova, Mikhail G. Divashuk, Anna V. Kiseleva, Alexandra I. Ershova, Alexey N. Meshkov, Oxana M. Drapkina

**Affiliations:** 1 National Medical Research Center for Therapy and Preventive Medicine, 101990 Moscow, Russia; blokhina0310@gmail.com (A.V.B.); kharlapmaria@yahoo.com (M.S.K.); marija.zaicenoka@gmail.com (M.Z.); anna.petukhova.96@gmail.com (A.V.P.); vostryakova.elizaveta@gmail.com (E.V.G.); azharikova89@gmail.com (A.A.Z.); divashuk@gmail.com (M.G.D.); sanyutabe@gmail.com (A.V.K.); alersh@mail.ru (A.I.E.); meshkov@lipidclinic.ru (A.N.M.); drapkina@bk.ru (O.M.D.); 2 Moscow Center for Advanced Studies, 123592 Moscow, Russia; 3 Faculty of Bioengineering and Bioinformatics, Lomonosov Moscow State University, 119991 Moscow, Russia; 4 All-Russia Research Institute of Agricultural Biotechnology, 127550 Moscow, Russia

**Keywords:** long QT syndrome, bradycardia, exome sequencing, genetic testing, *HCN4*, *SCN5A*, in silico variant effect prediction

## Abstract

**Background:** Genetic testing for long QT syndrome (LQTS) is straightforward in many families; however, in severe and complex cases, a single disease-causing variant may not be enough to explain all clinical features. In such cases, the search for genetic modifiers may be beneficial for precise diagnosis and management. **Case presentation:** We describe a three-generational family affected with clinically heterogeneous LQTS type 3 and bradycardia in which a novel missense variant p.V642M in *HCN4* was identified in addition to the known pathogenic variant p.E1784K in *SCN5A*. We performed the detailed clinical investigation of the family and a deep in silico analysis of the discovered variants, showing the causal role of a new *HCN4* variant in sinus bradycardia and its possible contribution to the phenotypic heterogeneity of LQTS type 3. **Conclusions:** This case is the first description of a functional variant in *HCN4* as a candidate modifier in LQTS type 3 and demonstrates the importance of analyzing additional genetic variations in families with complex LQTS phenotypes.

## 1. Introduction

Long QT syndrome (LQTS), affecting about 1:2500 individuals [1], is one of the most well-studied inherited disorders [2]. Three major types of LQTS have been extensively described [3], and the genotype–phenotype correlations for the causal variants in *KCNQ1*, *KCNH2*, and *SCN5A* genes are well known [4]. Thus, in many cases, the identification of a damaging variant in one of three aforementioned genes is enough to establish the diagnosis, stratify cardiac risks, and determine the treatment strategy and follow-up tactics both in probands and their relatives [5]. However, in a number of cases, genetic diagnosis and further family management may be complicated because the clinical features between the relatives are highly heterogeneous or remain unexplained [6,7]. The genetic mechanisms of such variability are still not fully investigated. First, the clinical spectrum of mutations in a single gene may be vast; the obvious example is *SCN5A*, associated with LQTS type 3. The *SCN5A* gene, encoding the vital cardiac sodium channel, is responsible for multiple clinically diverse heart rhythm disorders [8,9]; in turn, LQTS type 3, caused by *SCN5A*-damaging variants, is difficult to manage [10] and is characterized by a wider spectrum of symptoms, including frequent bradycardia [11,12] and the higher lethality of arrhythmic events than in other LQTS subtypes [13]. Another understudied reason for LQTS heterogeneity is the influence of modifier genetic variants [14,15], of which rare modifiers and their interaction with disease-causing variants are especially interesting. The genes encoding minor ion channels and other proteins of the cardiac conduction system are the most obvious candidates for the search of LQTS modifiers. One such gene is *HCN4*, which encodes the hyperpolarization-activated channel and is implicated into the development of sinus bradycardia [16,17]. The *HCN4* gene is functionally close to *SCN5A,* and there is a known overlap between *HCN4*- and *SCN5A*-related conditions [18] and suggestions on the possible modifying role of *HCN4* in Brugada syndrome [19], which is also a *SCN5A*-related disease. However, to our knowledge, the possible involvement of *HCN4* in LQTS type 3 modification has not been described yet. In the present study, we aimed to characterize a novel variant in *HCN4* recently reported by our team [20] and investigate its putative impact on the disease course in a family with clinically heterogeneous LQTS type 3. For this purpose, we provide the detailed clinical presentation of the family and an in silico functional study of genetic findings.

## 2. Materials and Methods

### 2.1. Clinical Investigation

Three generations of a family with a referral diagnosis of LQTS were examined at the Expert Arrhythmology Center and the Cardiology Department of the National Medical Research Center for Therapy and Preventive Medicine (NMRC TPM), Moscow, Russia. A clinical cardiological investigation was performed according to the European recommendations for the management of patients with LQTS [21], including a 12-lead electrocardiogram (ECG), 24 h Holter monitoring (24HM), and echocardiography (ECHO). Pre-admission clinical data were retrospectively analyzed from the patients’ medical records.

QT interval assessment was performed using a 12-lead ECG recorded under resting conditions. The QT interval was measured manually in lead V5 or V6 from the beginning of the QRS complex to the end of the T wave, defined by the tangent method. Rate correction was performed using the Bazett formula; the Framingham method was used for heart rates less than 60 and greater than 100 beats per minute.

The study was conducted in accordance with the Declaration of Helsinki and approved by the Review Board of the Biobank of the NMRC TPM. All participants (or their legal representatives, if applicable) gave their written informed consent to the study. The patients’ blood samples, used for genetic testing, were stored in the Biobank of the NMRC TPM.

### 2.2. Genetic Testing

**Figure 1:**
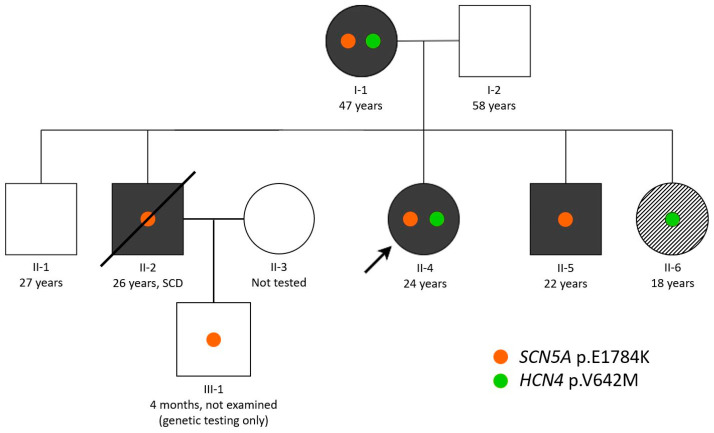
Pedigree of the studied family (ages are given at the time of genetic testing). The proband is marked with the arrow. Colored figures represent the affected family members as follows: black color—the long QT phenotype; diagonal black–white shading—bradycardia without QT prolongation. The crossed-out figure corresponds to the deceased family member. Genetic variants found in the family are shown with colored dots.

DNA was isolated from venous blood with the QIAamp DNA Blood Mini Kit (Qiagen, Hilden, Germany); the following quality control was performed on Qubit 4.0 fluorimeter (Thermo Fisher Scientific, Waltham, MA, USA). All available family members, except the proband’s father and infant nephew (I-2 and III-1 in Figure 1, respectively), underwent genetic testing by whole exome sequencing (WES). The validation of WES findings and the cascade screening of family members I-2 and III-1 was performed by Sanger sequencing.

The WES analysis was made with the IDT-Illumina TruSeq DNA exome libraries on NextSeq 550 (Illumina, San Diego, CA, USA) as per the manufacturer’s protocol. Sequencing reads were aligned to the GRCh38 version of human reference genome with bwa-mem [22]; nucleotide variants were called using GATK 4.2 HaplotypeCaller [23] and annotated using Ensembl Variant Effect Predictor (VEP) [24]. Variants with a minor allele frequency (MAF) less than 3% in the gnomAD database v3.1.2 [25] were selected for clinical interpretation, which we performed in accordance with the LQTS-specific recommendations based on standard ACMG/AMP guidelines [4,26].

For Sanger sequencing, we used the ABI PRISM BigDye Terminator Reagent Kit v3.1 or 1.1 (Thermo Fisher Scientific, Waltham, MA, USA) and analyzed the samples on the Applied Biosystems 3500 Genetic Analyzer (Thermo Fisher Scientific, Waltham, MA, USA) according to the manufacturer’s protocol.

### 2.3. In Silico Functional Analysis

Variant pathogenicity predictions were assessed by a number of tools. BayesDel addAF [27], FATHMM [28], AlphaMissense [29], MutationTaster2 [30], MetaSVM, MetaLR [31], PROVEAN [32], and REVEL [33] scores and predictions were retrieved from dbNSFP v4.7 [34,35] via Ensembl VEP. Other pathogenicity predictions were retrieved from the appropriate web-servers: PolyPhen-2 [36] (http://genetics.bwh.harvard.edu/pph2, accessed on 3 February 2025), ESM1b [37,38] (https://huggingface.co/spaces/ntranoslab/esm_variants, accessed on 3 February 2025), and MutPred2 [39] (http://mutpred.mutdb.org, accessed on 5 February 2025).

Evolutionary conservation was assessed with three tools—GERP++ [40], phyloP [41], and SiPhy [42]. The GERP++ scores range from −12.30 to 6.17; the larger the score, the more conserved the site is. GERP++ scores above 4 are often considered to be deleterious, though decreasing the threshold might increase the number of putatively deleterious mutations identified [43]. The phyloP scores were retrieved from the multiple alignment of 100 vertebrate species, with scores ranging from −20 to 10 and with larger scores being associated with more evolutionary conserved sites. The SiPhy log odds scores were constructed on 29 mammalian genomes, with the scores ranging from 0 to 37.97 and with larger scores indicating more conserved sites.

MutPred2 [39] and HOPE [44] were further used to infer possible structural changes due to missense variants.

MuPro [45], I-Mutant 2.0 [46], and DDMut [47] were used to assess the impact of amino acid substitutions on protein stability. These methods evaluate the change in thermodynamic free energy (ΔΔG) between wildtype and mutant proteins, with ΔΔG values less than zero indicating the destabilizing effect of amino acid substitution. DDMut requires protein structures as input. In the case of HCN4 protein, the full wild-type protein structure could be obtained from the Protein Data Bank (PDB) (PDB ID: 3OTF). In the case of Nav1.5, encoded by *SCN5A*, no wild-type protein structure was available in PDB, so the AlphaFold protein structure database [48] (https://alphafold.ebi.ac.uk, accessed on 3 February 2025) was used to obtain the protein structure.

## 3. Results

The pedigree of the studied family is shown in Figure 1.

### 3.1. Clinical Presentation of the Proband

A 24-year-old female proband (II-4, Figure 1) was first admitted to the NMRC TPM at the age of 18. She had a history of frequent syncope lasting up to one minute since the age of two weeks. From the age of six, she reported fatigue and weakness, and the syncopal episodes were typically stress-induced.

At the age of 11, an ECG showed a prolonged QT interval (a corrected QT interval (QTc) of up to 470 milliseconds (ms)). Based on the clinical features and electrocardiographic measurements, LQTS type 3 was suspected. The beta-blocker atenolol and the sodium channel blocker allapinin (lappaconitine hydrobromide [49]) were prescribed.

At the age of 13, 24HM revealed conduction disturbances, including atrioventricular and sinoatrial blocks, as well as the episodes of ventricular fibrillation less than 30 s. For the primary prevention of sudden cardiac death (SCD), a Virtuoso DR D164AWG cardioverter defibrillator (ICD) was implanted, and the same antiarrhythmic therapy was continued.

At the age of 16, the ICD system recorded five appropriate discharges due to sustained ventricular tachycardia. An ECG and 24HM showed a pacemaker rhythm with active atrial stimulation and a QTc of up to 549 ms with pronounced sinus bradycardia.

**Figure 2:**
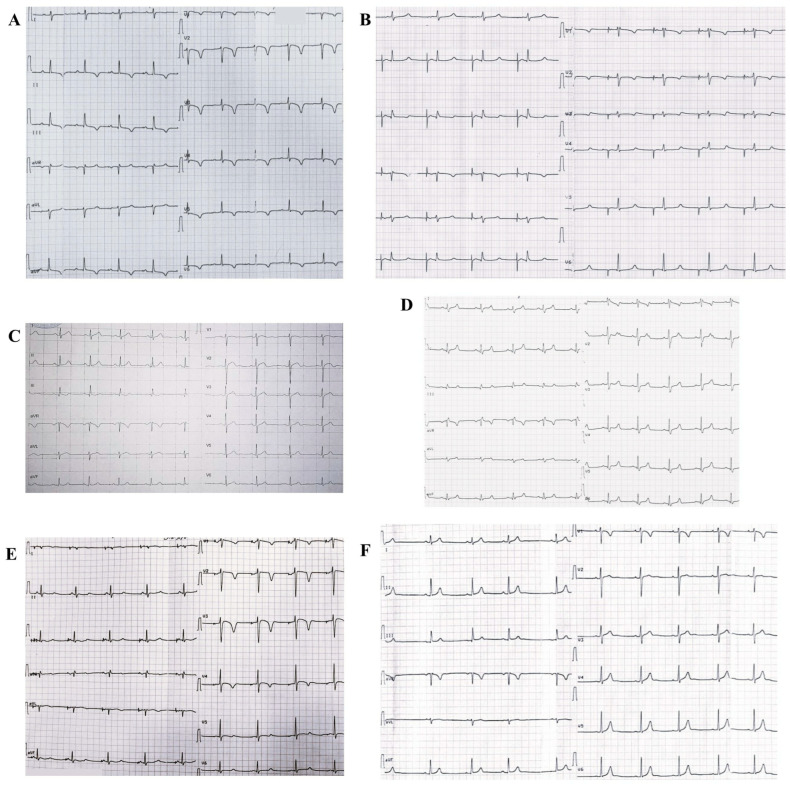
Patients’ electrocardiograms (ages are given at the time of ECG examination). (**A**) Proband (21 years): atrial paced rhythm with native ventricular conduction, heart rate—60 bpm. First-degree AV block. QT = 500 ms, QTc = 500 ms; (**B**) Proband’s mother (47 years): atrial paced rhythm with native ventricular conduction, heart rate—50 bpm. Incomplete right bundle branch block. QT = 540 ms, QTc = 509 ms; (**C**) Proband’s brother II-1 (27 years): sinus rhythm with a heart rate of 61 bpm. QT = 378 ms, QTc = 381 ms; (**D**) Proband’s brother II-2 (26 years): sinus bradyarrhythmia with a heart rate of 51–57 bpm. Right bundle branch block. QT = 500 ms, QTc = 483 ms; (**E**) Proband’s brother II-5 (19 years): atrial paced rhythm with native ventricular conduction, heart rate—50 bpm. QT = 560 ms, QTc = 529 ms; (**F**) Proband’s sister (18 years): sinus bradycardia with a heart rate of 51 bpm. Sinus arrhythmia. QT = 400 ms, QTc = 373 ms.

At the age of 17, the ICD system was replaced with a Teligen 100. The patient then underwent regular follow-up in our cardiology department, during which no ventricular arrhythmias or ICD discharges were observed. The maximum QTc of 610 ms was observed at the age of 24, according to the ECG, and no abnormalities were found on ECHO. The proband’s ECG is presented in Figure 2A.

### 3.2. Phenotypic Cascade Screening of the Relatives

The proband’s mother (I-1, Figure 1), aged 47, had presyncopal episodes since adolescence. At the age of 34, she was diagnosed with LQTS type 3 (maximum QTc 620 ms by ECG). At the age of 37, she was hospitalized for an episode of severe weakness and dizziness. After 24HM detected a transient third-degree AV block, a pacemaker was implanted in DDD mode. Since then, she has undergone regular follow-ups at the cardiology department of the NMRC TPM. She was prescribed metoprolol succinate therapy, and no life-threatening arrhythmias were recorded according to the 24HM data. The ECG is presented in Figure 2B.

The proband’s father (I-2, Figure 1), aged 58, and the proband’s eldest brother (II-1, Figure 1), aged 27 and having a QTc of 381 ms (Figure 2C), have no cardiac rhythm or conduction disturbances.

The proband’s brother (II-2, Figure 1) died of SCD at the age of 26 following a ventricular fibrillation storm. During cascade screening of the proband’s relatives, LQTS type 3 was diagnosed at the age of 12. At the age of 13, he had two short syncopes caused by orthostasis with spontaneous recovery. Since then, he had been receiving atenolol and allapinin, and the syncope had not repeated. At the age of 16, sinus bradycardia, rhythm pauses up to 2160 ms, and transient first-degree AV block were detected on ECG and 24HM. No structural or functional changes were found on ECHO. At the same age, a long-term ECG monitor, REVEAL XT 9529, was implanted. At the age of 17, 24HM indicated pronounced sinus bradycardia with pauses of up to 2128 ms; the maximum QT interval was 656 ms, while QTc did not reach 500 ms due to bradycardia. Then, during follow-up, syncope developed after a blood test, with recovery of cardiac activity after resuscitation. REVEAL and 24HM recorded sinus bradycardia and asystole with a maximum duration of up to 16,121 ms. Because of the high risk of SCD, the Biotronic Lumax 340 DR-T SN ICD was implanted. The patient underwent follow-up during which no ventricular arrhythmias, syncopes, or episodes of ICD discharges were observed. The ECG is presented in Figure 2D.

The proband’s younger brother (II-5, Figure 1), aged 22, was diagnosed with LQTS type 3 at the age of 8 (maximum QTc is 580 ms by ECG), and 24HM revealed pronounced sinus bradycardia. Atenolol and allapinin were initiated at the age of 10 due to episodes of presyncope. REVEAL was implanted at the age of 12 and has reported no ventricular arrhythmias since. The primary implantation of the ICD Taligen 100 was performed at the age of 16. Later, at the age of 21, four episodes of ICD discharges were observed, usually starting at night. During the follow-up, the dose of atenolol was increased, and no life-threatening arrhythmias were recorded. The ECG is presented in Figure 2E.

The proband’s sister (II-6, Figure 1), aged 18, was diagnosed with bradyarrhythmia in childhood (minimum heart rate 51 bpm according to ECG (Figure 2F)) without syncopal episodes. Given the family disease history and the known high incidence of bradyarrhythmia in LQTS type 3 [11,12], bradycardia was initially considered a possible manifestation of familial LQTS. At the age of 17, she was hospitalized for an episode of chest pain during physical exertion. During that hospitalization, 24HM showed a normal mean heart rate, and no QTc prolongation was revealed (maximum QTc of 410 ms). No abnormalities were found on ECHO.

The proband’s nephew (III-1, Figure 1), aged four months at admittance, was not clinically examined due to his infancy. We collected blood samples from this patient for cascade genetic testing.

### 3.3. Genetic Testing Results

The genetic testing of the proband revealed a known pathogenic missense variant in *SCN5A* (NM_001099404.2:c.5350G>A; NP_001092874.1:p.E1784K). This variant is the most common causal finding in LQTS type 3 [50], proven to be damaging by functional studies [51,52]. The cascade genetic screening of the proband’s relatives showed the presence of the p.E1784K variant in the affected mother and two brothers (II-2 and II-5, Figure 1) and, later, in the infant son of patient II-2. The proband’s younger sister (II-6) affected with sinus bradycardia, however, lacked this variant, as well as the unaffected family members—the proband’s father and eldest brother (II-1).

In addition to the *SCN5A* finding, we discovered a previously undescribed missense variant in the bradycardia-associated gene *HCN4* (NM_005477.3:c.1924G>A; NP_005468.1:p.V642M). This variant was also found in the proband’s mother and younger sister, thus co-segregating with the bradycardia phenotype in this family. Sanger chromatographs demonstrating the family segregation of two reported findings are presented in Figure S1.

### 3.4. In Silico Functional Analysis of HCN4 and SCN5A Variants

To evaluate the functional consequences of the genetic findings in the studied family, we performed an extensive in silico assay, which has been proven to be an efficient and cost-effective approach to the analysis of missense variants [53]. We analyzed both the previously uncharacterized *HCN4* p.V642M variant and the *SCN5A* p.E1784K variant to evaluate both variants simultaneously.

Variant pathogenicity was predicted with 12 in silico tools (PolyPhen-2, BayesDel addAF, ESM1b, FATHMM, AlphaMissense, CADD [54], MutationTaster2, MetaSVM, MetaLR, PROVEAN, MutPred2, and REVEL). In the case of *HCN4* p.V642M, all 12 tools (100%) predicted that the variant is damaging. In the case of *SCN5A* p.E1784K, 11 of the 12 instruments (91.7%) predicted the variant to be damaging, while PolyPhen-2 predicted it to be benign (Table 1).

The evolutionary conservation estimates retrieved for both variants suggest that both positions are rather conserved. On the other hand, *SCN5A* p.E1784K was shown to be more conserved than *HCN4* p.V642M by all three tools used—GERP++, phyloP, and SiPhy (Table 1).

MutPred2 additionally predicted possible changes caused by amino acid substitutions. Authors [39] suggest using Pr > 0.25 to assume the possible effect on property, although values lower than 0.25 can also be considered. MutPred2 suggests that *SCN5A* p.E1784K would result in the gain of relative solvent accessibility, gain of ubiquitination at amino acid substitution site, altered ordered interface, altered transmembrane protein (significant *p*-value, but Pr < 0.25), and, possibly, altered metal binding (borderline *p*-value 0.03, Pr < 0.25). Previous reports of functional analysis indicate that *SCN5A* p.E1784K leads to faster current decay and altered gating [51,55], which may be directly connected with alterations in the resulting protein structure. As for *HCN4* p.V642M, according to MutPred2, this variant would lead to a loss of relative solvent accessibility and the alteration of the transmembrane protein (significant *p*-value, but Pr < 0.25).

Another three tools were used to evaluate the amino acid substitution effect on protein stability—MUPro, I-Mutant 2.0, and DDMut. All tools agree that both substitutions lead to less stable protein. DDMut predicted very small ∆∆G changes (−0.01), which might be caused by the use of the AlphaFold-predicted structure instead of the PDB experimental structure.

## 4. Discussion

Here, we present the family of three generations harboring the established LQTS-causing variant in *SCN5A* and an additional previously undescribed rare variant in *HCN4*. The family disease phenotype is remarkable due to the prominent bradycardia, which we observed in all affected family members. Previous studies of *SCN5A* p.E1784K describe frequent bradycardia in the carriers [51]; at the same time, the *HCN4* gene is known to be associated with sinus rhythm disorders and bradyarrhythmia [56]. Thus, the presence of an additional variant p.V642M in *HCN4* may also contribute to the bradyarrhythmic phenotype of the affected family members. We tried to define this contribution through the analysis of the functional consequences of our genetic findings.

The increase in the severity of an LQTS phenotype in the patients that harbor rare variants additional to the disease-causing mutations has been described in some studies [20,57,58], but the mechanism and the extent of such a modification is hard to evaluate. In recent years, emerging innovations such as patient-specific induced pluripotent stem cells (iPSC) and CRISPR/Cas genome editing have enhanced our opportunities in deciphering the functional effect of specific rare variants [59,60], but such methods require substantial laboratory facilities and highly specific expertise. When innovative technologies are unavailable or unaffordable, in silico computational predictions of variant effects may be of help and facilitate the characterization of unknown variants [61]. In our case, the results of the deep in silico analysis of *HCN4* p.V642M strongly suggest the deleterious effect of this variant on the protein, and the presence of severe bradycardia in the proband’s sister, who is genotype-negative for LQTS type 3 and harbors only the *HCN4* p.V642M substitution, testifies to the causal role of this variant in sinus bradycardia.

However, the mechanism of the *HCN4* p.V642M impact on the bradyarrhythmic phenotype of the *SCN5A* p.E1784K carriers remains unclear. Given the contribution of the “funny” current generated by the HCN4 channel to the spontaneous pacemaker activity of sinoatrial cardiomyocytes [62,63], we can speculate that in the carriers of *HCN4* p.V642M, pacemaker activity is depleted due to the HCN4 protein damage. This, in turn, may aggravate the *SCN5A* p.E1784K-caused bradycardia; however, analyzing the outcomes in the studied family, we cannot evaluate to what extent the *HCN4* variant works as the “second hit” on the heart rate since the most severe outcome, namely SCD, occurred in the patient II-2 without the *HCN4* variant. Nevertheless, it is of note that the SCD outcome did not quite correlate with the overall severity of the phenotype, since the most severely affected member of the studied family is the proband with frequent recurrent syncopal episodes since infancy, who harbors both reported variants. Regarding the immediate cause of SCD in the proband’s brother, we cannot exclude the impact of sex-specific mechanisms that are known to have a protective effect against SCD on women [64], particularly regarding the potential impact of sex hormones on *HCN4* function within pacemaker cells [65], which is not fully studied yet.

Based on all the above, we interpret the *HCN4* p.V642M finding as causal for bradycardia in patient II-6 and suggest its contribution to the arrhythmogenic landscape in LQTS-carrying relatives, considering the co-participation of *HCN4* and *SCN5A* in sinus node functioning [66]. Nevertheless, further functional studies are needed to confirm these suggestions.

### Limitations of the Study

All available retrospective patient data were analyzed as fully as possible. However, the primary implantation of cardiac resynchronization devices was performed before the admission to the NMRC TPM, and pre-intervention ECGs were not available for analysis; thus, we cannot accurately assess the pre-treatment severity of bradycardia. The conclusion of the damaging role of *HCN4* p.V642M was made based on the results of the in silico variant effect prediction and clinical investigation of patient II-6. Although an in vitro functional study would provide us with a higher level of evidence, we were not able to conduct any.

## 5. Conclusions

In the family with a complex LQTS type 3 phenotype, the combination of advanced genetic testing and the in silico functional study helped to clarify the genetic basis of the observed rhythm disorders, including a novel protein-damaging variant in *HCN4*, and to establish the causal role of this novel variant in sinus bradycardia. This strategy allowed us both to establish the absence of LQTS in the proband’s sister and to clarify the clinical heterogeneity among relatives. This case illustrates the importance of analyzing additional genetic variations in families with complex LQTS phenotypes and underscores that in difficult cases, an expanded genetic testing approach may be valid not only for research purposes but also for correct diagnosis and management.

## Figures and Tables

**Table 1:** Results of deep in silico analysis of *SCN5A* p.E1784K and *HCN4* p.V642M variants.

Software Tool	*SCN5A* p.E1784K	*HCN4* p.V642M
Pathogenicity Predictions		
PolyPhen-2, prediction (score) [36]	Benign (0.43)	Probably damaging (0.99)
BayesDel addAF, prediction (score) [27]	Damaging (0.49)	Damaging (0.31)
ESM1b, prediction (score) [37]	Damaging (−12.96)	Damaging (−13.81)
FATHMM, prediction (score) [28]	Damaging (−3.87)	Damaging (−3.49)
AlphaMissense, prediction (score) [29]	Likely pathogenic (0.87)	Likely pathogenic (0.94)
CADD, prediction (score) [54]	Top 1% deleterious (26.0)	Top 0.1% most deleterious (31.0)
MutationTaster2, prediction (score) [30]	Disease causing (0.99)	Disease causing (0.99)
MetaSVM, prediction (score) [31]	Damaging (1.05)	Damaging (0.94)
MetaLR, prediction (score) [31]	Damaging (0.92)	Damaging (0.89)
PROVEAN, prediction (score) [32]	Damaging (−3.57)	Damaging (−2.79)
MutPred2, prediction (score) [39]	Pathogenic (0.85)	Pathogenic (0.60)
REVEL, prediction (score) [33]	Pathogenic (0.91)	Pathogenic (0.85)
Conservation		
GERP++, score [40]	4.82	3.68
PhyloP vertebrate, score [41]	6.14	4.04
SiPhy, score [42]	18.09	9.45
Structural and functional properties		
MutPred2	Gain of relative solvent accessibility (Pr = 0.36|*p* = 9.4 × 10^−4^); Gain of Ubiquitylation at E1784 (Pr = 0.30|*p* = 5.3 × 10^−4^); Altered ordered interface (Pr = 0.28|*p* = 0.04); Altered transmembrane protein (Pr = 0.18|*p* = 8.0 × 10^−3^); Altered metal binding (Pr = 0.16|*p* = 0.03)	Loss of relative solvent accessibility (Pr = 0.27|*p* = 0.02); altered transmembrane protein (Pr = 0.19|*p* = 6.0 × 10^−3^)
HOPE [44]	Wild-type residue is charged negatively, while mutant residue is charged positively Mutant residue is bigger than wild-type residue Residue is located on the surface; thus, the mutation might disturb interactions	Mutant residue is bigger than wild-type residue Residue is located on the surface; thus, the mutation might disturb interactions
Effects on protein stability		
MUPro, Effect on stability (∆∆G, kcal/mol) [45]	Decrease (−1.33)	Decrease (−0.98)
I-Mutant 2.0, Effect on stability (∆∆G, kcal/mol) [44]	Decrease (−1.13)	Decrease (−1.65)
DDMut, Effect on stability (∆∆G, kcal/mol) [47]	Decrease (−0.01)	Decrease (−0.38)

## Data Availability

The data used in this study, including individual genotype information, cannot be publicly disclosed according to the rules of the Ethics Committee of the National Medical Research Center for Therapy and Preventive Medicine. Deidentified data will be provided upon reasonable request by the corresponding author, Anna Bukaeva (annbukaeva@gmail.com) or by the researcher of the Clinomics laboratory, Anastasia Blokhina (blokhina0310@gmail.com) or by the Ethics Committee of the National Medical Research Center for Therapy and Preventive Medicine (phone number +74995536810, secretarynec@gnicpm.ru). Proposals will be reviewed and approved by the investigators, local regulatory authorities, and the Ethics Committee of the National Medical Research Center for Therapy and Preventive Medicine. Once the proposal is approved, data can be transferred through a secure online platform after signing a data access agreement and a confidentiality agreement.

## Supplementary Materials

The following supporting information can be downloaded at https://www.mdpi.com/article/10.3390/biomedicines13041008/s1, Figure S1: Sanger chromatographs of genetic findings in the studied family.
