# Supplementary figures and images for: A Novel Bradycardia-Associated Variant in HCN4 as a Candidate Modifier in Type 3 Long QT Syndrome: Case Report and Deep In Silico Analysis
Source: Biomedicines. 2025 Apr 21;13(4):1008. doi: 10.3390/biomedicines13041008 (PMC12025296; doi:10.3390/biomedicines13041008)

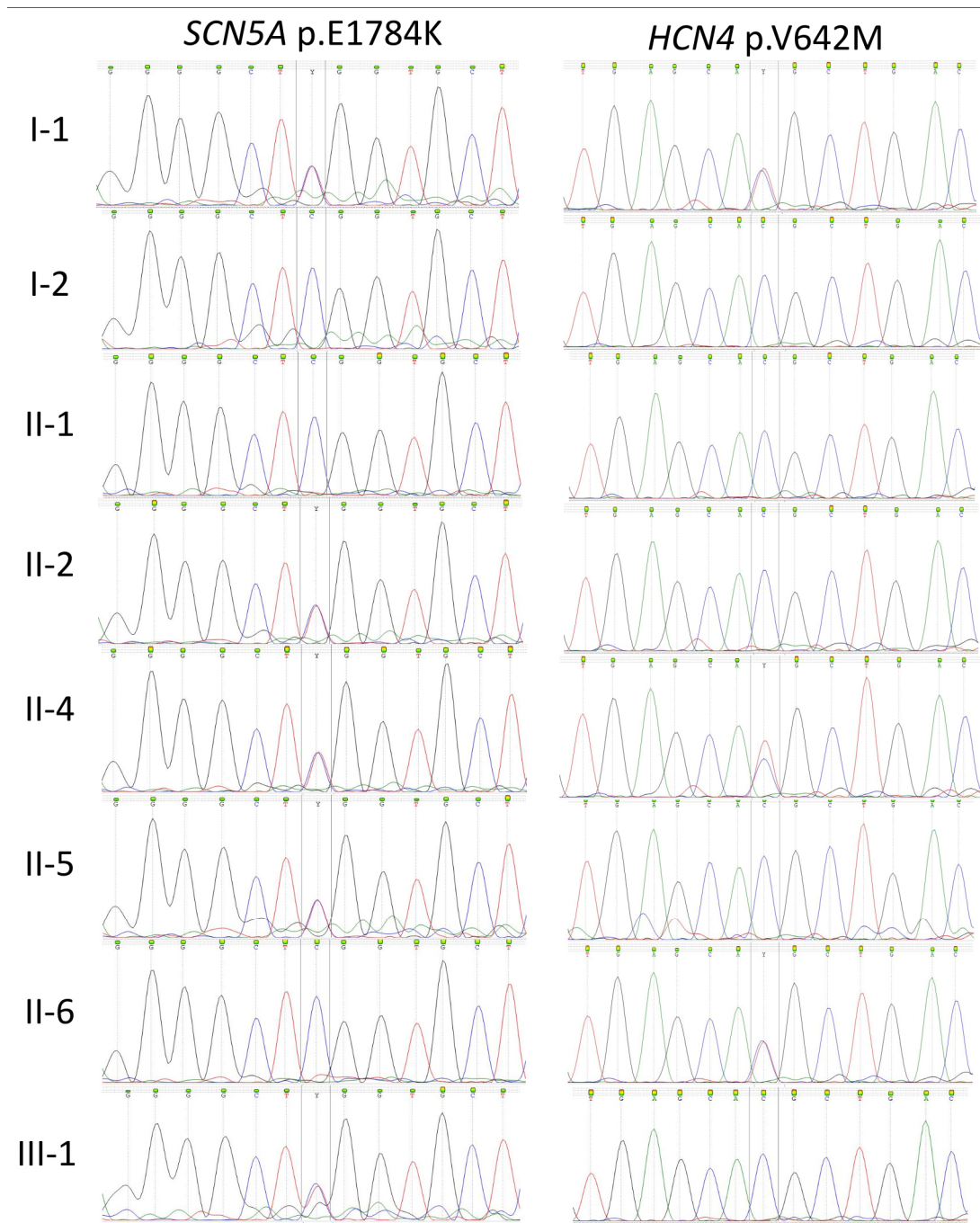

**Figure S1.** Sanger chromatographs of genetic findings in the studied family.

Supplement: Supplementary file 1 [file biomedicines-13-01008-s001.zip › biomedicines-3536849-supplementary.pdf]
